# Sulfur isotope analysis for representative regional background atmospheric aerosols collected at Mt. Lulin, Taiwan

**DOI:** 10.1038/s41598-019-56048-z

**Published:** 2019-12-23

**Authors:** Chuan-Hsiung Chung, Chen-Feng You, Shih-Chieh Hsu, Mao-Chang Liang

**Affiliations:** 1 Department of Earth Sciences, National Cheng Kung University, Tainan, Taiwan; 2 Research Center for Environmental Changes, Academia Sinica, Taipei, 11529 Taiwan; 3 Institute of Earth Sciences, Academia Sinica, Taipei, 11529 Taiwan

**Keywords:** Atmospheric chemistry, Environmental monitoring, Environmental impact

## Abstract

Air pollution resulted from fossil fuel burning has been an environmental issue in developing countries in Asia. Sulfur-bearing compounds, in particular, are species that are regulated and monitored routinely. To assess how the species affect at local and global scales, regional background level has to be defined. Here, we report analysis of sulfur isotopes in atmospheric sulfate, the oxidation end product of sulfur species, in particulate phase collected at the Lulin observatory located at 2862 m above mean sea level in 2010. The averaged sulfate concentration for 44 selected samples is 2.7 ± 2.3 (1-σ standard deviation) μg m^−3^, and the averaged δ^34^S is 2.2 ± 1.6‰, with respect to the international standard Vienna Canyon Diablo Troilite. Regardless of the origins of air masses, no noticeable difference between the low-altitude Pacific and high-altitude free troposphere sulfate aerosols is observed. Also, no identifiable seasonal cycle in seen. Correlation analysis with respect to coal burning tracers such as lead and oil industry tracers such as vanadium shows sulfate concentration is in better correlation with vanadium (R^2^ = 0.86, p-value < 0.001) than with lead (R^2^ = 0.45, p-value < 0.001) but no statistically significant correlation is found in δ^34^S with any of physical quantities measured. We suggest the sulfate collected at Lulin can best represent the regional background level in the Western Pacific, a quantity that is needed in order to quantitatively assess the budget of sulfur in local to country scales.

## Introduction

Sulfur is ubiquitous in natural environments and in the atmosphere is present primarily either as sulfate in aerosol/aqueous phases or as OCS and SO_2_ in gas phase. Sulfur isotopic compositions vary with sources and cycling pathways, thus have received tremendous attention as key tracers in geochemical, biological, and atmospheric processes^1–6^. Sulfur isotopes have also been used to investigate sources and chemical evolution processes of atmospheric aerosols^7–9^. The main source materials of atmospheric sulfur include volcanic sulfur, marine sulfate/sulfide, fossil fuel, and sulfide ores. Despite the wide distribution of stable sulfur isotope ratios (δ^34^S) in these materials (−50‰ to +50‰), the main sulfur emissions within a specific regional reservoir possess distinctive characteristics of sulfur isotopic values^10–14^. Table 1 summarize the range of δ^34^S values reported in the literature, along with the value determined in this work (see below).

**Table 1:** The sulfur isotopic values (δ^34^S) of sulfur-bearing species for known emission sources.

Source	δ^34^S_VCDT_ (‰)	Reference
**Anthropogenic sources**		
North China Coal	−3.9 to 11.2; avg: 3.7	^46^
South China Coal	−7.5 to 5.4; avg: −0.3	^46^
Chinese Crude Oil	7.2 to 24.2; avg: 15.2	^47^
Chinese Light Diesel Oil	13.7	^47^
Chinese Heavy Fuel Oil	20.6	^47^
Russian Heavy Fuel Oil	1.1	^47^
Malaysian Crude Oil	4.1 to 4.4; avg: 4.3	^47^
Brunei Crude Oil	3.7	^47^
Australian Crude Oil	6.8 to 8.4; avg: 7.6	^47^
Iranian Crude Oil	−2.6	^47^
UAE Crude Oil	−10.3 to −4.3; avg: −6.3	^47^
Saudi Arabian Crude Oil	−8.8 to −0.7; avg: −4.8	^47^
Omani Crude Oil	10.4	^47^
Japanese Crude Oil	10.8	^47^
Chinese Atmosphere(Summer)	−4.0 to 6.0	^45^
Chinese Atmosphere(Winter)	−2.2 to 6.4	^45^
Japanese Atmosphere(Summer)	−1.6	^45^
Japanese Atmosphere(Winter)	−1.2	^45^
Chinese Atmosphere(Mount Wuyi)	0.9 to 4.8	^49^
Chinese Atmospheric sulfate(Guangzhou)	4.2 to 7.2	^49^
Sulfate aerosols from Beijing China(Spring)	4.4 to 9.2; avg: 6.4	^7^
Sulfate aerosols from Beijing China(Summer)	3.4 to 7.0; avg: 5	^7^
Sulfate aerosols from Beijing China(Autumn)	5.0 to 9.4; avg: 6.8	^7^
Sulfate aerosols from Beijing China(Winter)	7.1 to 11.3; avg: 8.6	^7^
**Volcanic emissions**		
High Temperature Primary Sulfates	1 to 9	^11^
Tropospheric Secondary Sulfates	0 to 15	^11^
Stratospheric Secondary Sulfates	−5 to 20	^11^
Sulfate aerosols from LLN	−1.0 to 8.2; avg: 2.2	This work

Oceanic Dimethyl sulfide has a value of δ^34^S around 21‰ (ref. ^13^).

Climatically identified to be the most significant aerosol species that gives negative radiative forcing^15^ (−0.4 ± 0.2 W m^−2^), atmospheric sulfate is produced primarily by aqueous phase chemical reactions (via oxidation chemistry with dissolved ozone and hydrogen peroxide) in cloud droplets and by dust particle-mediated gas phase chemistry (via first oxidation of SO_2_ with hydroxyl radicals followed by subsequent condensation and heterogeneous chemistry) on pre-existing particles (e.g.,^16–21^). In addition to anthropogenic inputs, volcanic eruptions also release a significant amount of sulfur-bearing gases into the atmosphere of the Earth. In a global perspective, major sources of SO_2_ include fossil fuel burning (~72%), biomass burning (~2%), marine dimethyl sulfide (~19%), and volcanic emissions (~7%)^22^. The last are the most relevant species concerning the climatic impact of volcanic activities. For example, Eyjafjallajökull, a volcano on southern Iceland, began to erupt on 14 April 2010. The volcanic ash and gases were ejected several kilometers into the atmosphere and transported over long distance. The ash was detected over the Netherland, Germany, Italy, and Greece^23,24^. The transport distance of volcanic aerosols is expected to be much longer. However, little observation has been made on the transport pathways. In particular, a recent new analysis showed volcanic emissions of SO_2_ during passive degassing are 23 ± 2 Tg/yr (ref. ^25^), comparable to the total SO_2_ emission from China^26^ (see below for anthropogenic emissions). In this paper, we present the concentration and sulfur isotopic composition in aerosol sulfate, in attempt to see how anthropogenic and natural emissions (such as the Eyjafjallajökull mentioned above) affect regional sulfate concentration in a regional scale in Asia.

In the last two decades or so, anthropogenic emissions have been shifted from the western countries like USA and Europe to China and southeast Asia, leading to significant regional shifts in radiative forcing and environmental impact (e.g.,^26–31^). Indeed, it has been documented that China alone contributes nearly a quarter of the global emission (e.g.,^30^), amounting to ~30 Tg SO_2_ per year, largely from coal burning (~90%)^26^; China shares slightly more than 50% of world coal consumption^32^. Though fuel gas desulfurization device in power plants is widely applied, the emission from industry remains, accounting for ~70% to total SO_2_ emission from China^26^. Total emission from other countries in Asia is about 20% that of China^30^. We then expect that the regional background sulfur emissions and sulfate concentrations are largely set by coal emissions from China. A recent study by Sakata *et al*.^33^, however, does not support this scenario; and instead they noted that sulfate concentrations in Japan (from the results derived from two coastal sites) are heavily influenced by oil industry in seasons other than the core winter (December, January, and February). The new isotope and concentration analysis presented in this work also shows that the China coal emission signals are not clearly seen (see below). See Table 1 for the isotopic values of sulfur (δ^34^S) for known emission sources.

## Sampling and Extraction

The aerosol samples were collected at Lulin Atmospheric Background Station (NOAA code: LLN; 120°52′25″E, 23°28′07″N, 2,862 m above mean sea level) during 2010. This site is located on the summit of Mt. Lulin in central Taiwan and considered as a clean air station with minimum influence of local pollution. At such high elevation in the free troposphere, the observatory is an ideal station for monitoring levels of pollutants and background traces in regional to global scales (e.g., see Hsu *et al*.^34^). The location of the site allows for studies of long-range transport of aerosols^34^. Aerosol samples were collected daily using high volume TSP collectors^34^ onto pre-baked (900 °C for ~5 h) PALL Pallflex tissue quartz filters (8″ × 10″), and stored at a temperature close to 0 °C during transportation. The average volume of air that passed through the TSP collectors in a day was 1700 ± 275 m^3^ (the quoted error bar refers to 1-σ standard deviation of the sampling volume variation). For isotopic sulfate analysis, 44 samples were selected, chosen based on their five-day back trajectories, to best represent air masses in the region. They were originated either over Pacific Ocean, continental low altitudes, or mid-troposphere. In addition, the selection was also based on the consideration of possible seasonal variations. As a result, about 2–4 samples per month were picked. The aerosol was then extracted by shredding 1/16 part of a filter paper placed in a sterilized centrifuge tube, containing 10 ml ultrapure Milli-Q water, kept in an ultrasonic bath for 60 min. The extracts were then filtered using syringe filters (Minisart 17 597-K, pore size 0.2 mm), and the sample stock solution was ready for subsequent sample preparation procedures. The selected samples and their analytical results are summarized in Table 2. Supporting data such as CO, O_3_, major ions, and metals are obtained and measured following the methods described by Ou-Yang *et al*.^35^ and Hsu *et al*.^36^; the data of CO and O_3_ are available in Guha *et al*.^37^

**Table 2:** Summary of the concentrations (in ng/m^3^) and δ^34^S (in ‰) of sulfate for the 44 selected samples. Ions and metals relevant to the work are also shown.

	Al	Pb	V	nc-V	NH_4_^+^	Na^+^	Ca^2+^	Cl^−^	NO_3_^−^	SO_4_^2−^	nss-SO_4_^2−^	δ^34^S_VCDT_
**sampling date**												
7-Feb^**^	283	1.20	0.53	0.43	275	51	21	45	264	536	523	3.94
8-Feb^**^	340	1.03	0.62	0.49	421	85	38	78	446	952	930	2.73
10-Mar^**^	465	6.21	1.04	0.87	2852	123	218	492	3704	5133	5102	1.75
11-Mar^**^	450	6.10	1.32	1.15	3861	178	220	743	6259	7220	7175	2.72
12-Mar^*^	167	1.87	0.52	0.45	1063	103	112	185	1554	2311	2285	3.86
22-Mar^*^	235	2.51	0.85	0.77	920	214	93	322	1505	2030	1976	4.20
23-Mar^*^	223	3.44	0.56	0.48	2011	88	113	367	2610	2937	2915	3.58
12-Apr^**^	196	N/A	0.56	0.49	769	87	61	106	1023	1447	1425	3.17
13-Apr^**^	223	1.56	0.50	0.41	459	49	80	65	646	1003	990	3.11
19-Apr^**^	450	1.60	1.12	0.96	787	107	130	124	821	2092	2065	2.02
20-Apr^**^	179	0.52	0.42	0.35	552	117	68	165	685	1332	1302	0.96
21-Apr^*^	117	1.99	0.23	0.19	216	18	34	32	122	752	747	3.59
2-May^**^	464	3.98	1.47	1.30	1728	203	355	157	1918	4606	4555	1.63
3-May^**^	1056	6.14	1.97	1.58	3355	265	623	526	4040	7797	7731	2.56
4-May^**^	769	4.62	1.58	1.29	2718	197	442	354	3090	6125	6076	2.18
18-May^**^	169	1.15	0.58	0.52	848	119	110	169	1274	1926	1896	0.69
19-May^**^	302	1.62	0.97	0.85	1262	158	170	173	1587	2693	2653	0.59
23-May^**^	382	4.58	1.12	0.98	2702	124	340	228	2479	5949	5918	1.49
24-May^*^	95	2.24	0.85	0.81	1586	92	123	235	2082	3338	3315	2.09
25-May^**^	46	0.39	0.17	0.15	273	33	23	115	324	455	447	1.08
7-Jun^**^	176	2.34	0.85	0.79	1126	100	118	155	990	3400	3375	1.68
8-Jun^**^	36	0.38	0.10	0.08	184	26	21	132	211	325	318	2.71
26-Jun^**^	36	1.56	0.16	0.14	345	54	41	139	250	740	727	1.53
27-Jun^*^	74	0.60	0.32	0.29	486	97	69	192	515	1263	1239	1.42
20-Jul^**^	27	0.24	0.09	0.08	297	13	31	113	227	383	380	2.00
21-Jul^**^	26	0.60	0.41	0.40	701	23	72	163	620	1831	1826	3.72
22-Jul^**^	27	0.65	0.53	0.52	946	24	74	129	724	2450	2444	3.53
25-Jul^*^	2	0.03	N/A	N/A	91	3	3	80	N/A	N/A	N/A	0.22
26-Jul^*^	10	0.22	0.02	0.01	213	5	8	98	108	122	120	8.21
27-Jul^*^	21	0.55	0.07	0.06	208	28	56	123	236	438	431	1.55
2-Sep^*^	5	0.14	0.07	0.07	238	28	12	145	115	352	345	−0.24
3-Sep^*^	20	0.39	0.20	0.19	358	18	26	127	285	831	827	−0.96
4-Sep^*^	19	0.69	0.51	0.50	976	44	58	235	823	2211	2200	1.61
3-Oct^*^	44	3.58	0.65	0.64	798	64	37	40	569	2441	2425	5.59
4-Oct^*^	20	1.25	0.20	0.19	293	13	19	N/A	233	805	801	2.98
12-Oct^*^	68	3.81	1.28	1.25	2632	65	62	146	1975	4372	4356	0.94
13-Oct^*^	83	5.00	1.42	1.39	2771	68	66	176	2132	4724	4707	1.18
21-Nov^**^	337	8.64	0.90	0.78	1036	161	177	96	1017	3095	3054	1.60
22-Nov^**^	571	7.08	1.03	0.82	688	180	238	81	686	1965	1920	1.94
23-Nov^**^	431	11.62	1.09	0.93	1042	226	202	159	1023	3714	3658	2.17
5-Dec^**^	997	42.54	2.51	2.13	2768	325	669	334	2739	10433	10351	0.35
6-Dec^**^	632	8.86	1.02	0.78	690	161	293	74	672	2368	2328	2.85
28-Dec^**^	142	8.74	0.55	0.49	541	182	94	159	659	2056	2010	3.20
29-Dec^**^	406	22.76	1.02	0.87	956	135	207	115	1128	4010	3976	0.21

The superscripts * and ** refer to the source regions (based on five-day back trajectory) below and above the sampling location at Lulin. nc-V stands for non-crustal vanadium. See text for details.

### Sulfur isotope analysis

Acids used in this study for sample digestion are high purity ones procured from JT Baker. Acids were diluted using Milli-Q water (MQW; resistivity 18.2 MΩ cm). SPEX (aqueous NH_4_SO_4_), used as the bracketing standard, was from SPEX CertiPrep Group, Metuchen, USA. PFA vials used in this work were cleaned using sequential cleaning of hot HNO_3_, HCl and MQW for >12 hour durations. Anion exchange resin (AG1X8; Cl form; 200–400 mesh; BIORAD labs, Richmond, USA) has been used for separation of sulfate from other matrix elements. The sulfur separation procedures were adopted from Das *et al*.^38^. All operations (cleaning and sample preparation) were done in CLASS-1000 laboratory, and column chemistry was performed within CLASS-10 working bench maintained at positive airflow.

Dissolution of IAEA S-1 standard was made using protocols following Craddock *et al*.^39^. Aliquots of IAEA S-1 solution and aerosol stock solution were evaporated to dryness on a 65 °C hotplate contained within a homemade clean box equipped with the filtered influx air and a venting system to reduce possible contamination from the surroundings. Then, 2–4 mL of 0.3 N HNO_3_ were added to re-dissolve all the dried material and the sulfur concentrations were measured by ICP-OES. Known amount of this solution was dried and taken in 0.028 M HNO_3_ to yield sulfur stock concentration of 8 µg mL^−1^ for subsequent column chemistry. The recovered sulfur (2 µg) is finally taken in 1 mL of 0.3 M HNO_3_ for isotopic measurements. All measurements of δ^34^S were done using the Thermo Neptune MC-ICPMS (Thermo Fischer Scientific, Germany) facility at the Isotope Geochemistry Laboratory at the National Cheng Kung University, Taiwan. δ^34^S measurements were made in the high resolution mode, similar to that of Craddock *et al*.^39^, to separate sulfur from major molecular interferences. Isotopic measurements are made at masses ^32^S, ^33^S and ^34^S (monitored at L1, C and H1 faradays cups, respectively), and sulfur isotopic ratios are determined on the low mass shoulder to avoid heavier molecular interferences from O_2_. (In this work, we limit our discussion to ^34^S. Because of precision for ^33^S, no measurable mass-independent effect is found for the samples reported in this work.) Contributions of isobaric interference from ^64^Zn^2+^ and ^68^Zn^2+^ to ^32^S and ^34^S, respectively, were found to be negligible. This was assessed by scanning an ultra pure solution of 50 ng g^−1^ Zn and monitoring the signal intensities at ^32^S and ^34^S and was found to be similar to that of the HNO_3_ solution. Standard-sample-standard bracketing was used to correct for instrumental mass bias using the SPEX standard. Peak centering was done with respect to the ^34^S mass scan. All measurements were taken at an integration time of 4 seconds and data acquisition was made for 48 measurements. Mean isotopic ratios of bracketing standard (SPEX) and samples evaluated by the Neptune software were used for calculating δ^34^S. Two blank tests were performed during the analytical session, the overall procedural blanks vary between 12–18 ng. Since bracketing standard were processed through columns similar to that of a sample, no additional procedural blank correction was needed. Typical 2-σ external measurement precision (relative to SPEX) ranged from 0.24–0.34‰; however, the expanded (propagated) uncertainty increased to 0.45‰ because of two normalizations (sample and SPEX; IAEA S-1 and SPEX) involved in converting to VCDT (Vienna Canyon Diablo Troilite) scale. In the following, the value of IAEA S-1 has been assumed to have δ^34^S of −0.3‰ relative to VCDT^40^.

δ^34^S of a sample relative to the VCDT scale is calculated using the following relation:

$${{\rm{\delta }}}^{34}{{\rm{S}}}_{{\rm{VCDT}}}^{{\rm{sam}}}=[\frac{1+{{\rm{\delta }}}^{34}{{\rm{S}}}_{{\rm{SPEX}}}^{{\rm{sam}}}}{1+{{\rm{\delta }}}^{34}{{\rm{S}}}_{{\rm{SPEX}}}^{{\rm{IAEA}}\,{\rm{S}}-1}}\times (1+{{\rm{\delta }}}^{34}{{\rm{S}}}_{{\rm{VCDT}}}^{{\rm{IAEA}}\,{\rm{S}}1})-1].$$

## Results and Discussion

Overall, the concentrations of major ions are highly variable, with [NH_4_^+^] = 1115 ± 988 ng m^−3^, [SO_4_^2−^] = 2674 ± 2271 ng m^−3^, and [NO_3_^−^] = 1264 ± 1263 ng m^−3^. Largely affected by wet deposition, the concentrations are lower in summer time (June-September) than the rest of the time of the year. In summer, daily precipitation is 1.9 ± 2.6 mm (air relative humidity is 93 ± 7%); in winter and spring, the value is 0.2 ± 0.4 mm (air relative humidity is 78 ± 19%). [NH_4_^+^] is 474 ± 346 and 1383 ± 1048 ng m^−3^, respectively, for summer and the rest of the time; [SO_4_^2−^] is 1196 ± 1048 and 3246 ± 2368 ng m^−3^; [NO_3_^−^] is 425 ± 298 and 1589 ± 1345 ng m^−3^. Strong temporal variability in winter and spring is closely associated with northeast Asia monsoon that significantly modifies the trajectories of air masses arriving at the sampling location. The phenomenon has been noted previously from the analysis of multiple isotope compositions of nitrate aerosols collected at the same location^37^.

**Figure 1:**
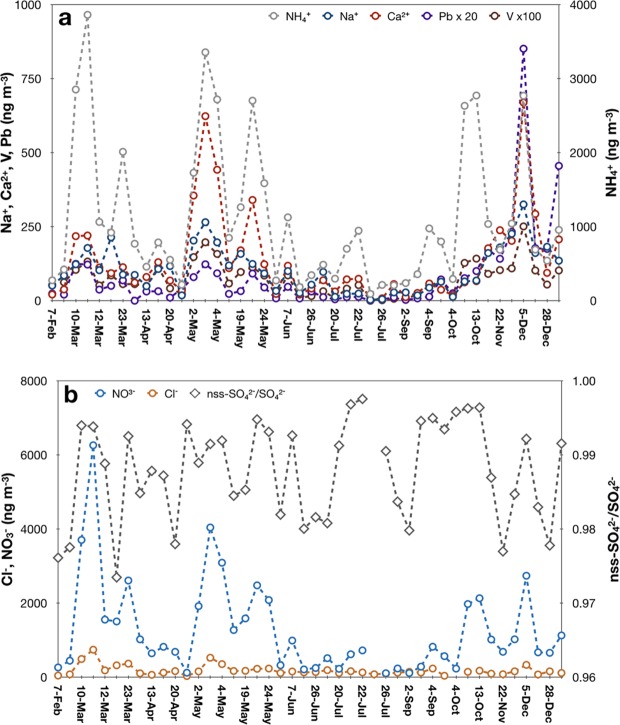
Time series of the concentrations (ng/m^3^) of major ions and metals (Na^+^, Ca^2+^, and NH_4_^+^, nss-SO_4_/SO_4_^2−^, NO_3_^−^, and Cl^−^. Pb and V). The data values are provided in Table 2.

In the region, there are three major sources of sulfur: ocean, oil industry (ship business), and coal burning. We examine them below. (The aforementioned natural sources such as volcanic emission from Eyjafjallajökull eruption as a major source of sulfate at LLN were not supported, because of good correlation between sulfate concentration and man-made trace metal levels such as vanadium and absence of correlation between δ^34^S and the other variables measured and analyzed in this work. See the analysis presented below for details.) Fig. 1 shows the time series of the observed major ions for the selected 44 aerosol samples. The single most important cation is NH_4_^+^, contributing 76 ± 8%, followed by Ca^2+^ (8 ± 4%), K^+^ (7 ± 5%), Na^+^ (6 ± 4%), and Mg^2+^ (3 ± 1%). The positively charged ions are balanced primarily by SO_4_^2−^, NO_3_^−^, and Cl^−^, with the first two contributing 90 ± 8%. SO_4_^2−^ is about a factor of 3 more important than NO_3_^−^; the former accounts for 67 ± 11% and the latter is 24 ± 6%. Cl^−^ contribution is variable at 10 ± 8%, with a maximal contribution of 40% appearing on July 26 when the highest δ^34^S value in sulfate is observed (see Table 2). Surprisingly the lowest sea salt anion contribution (the fraction of Cl^−^) to the selected occurring on October 3 corresponds to the second largest δ^34^S value measured. The data shows that the δ^34^S values of sulfates do not follow the fraction of sea salts in the collected aerosols, suggesting that oceanic sulfur contribution to the sulfate observed at LLN is variable but is not likely to be the major source. Further analysis for the other two sources follows.

**Figure 2:**
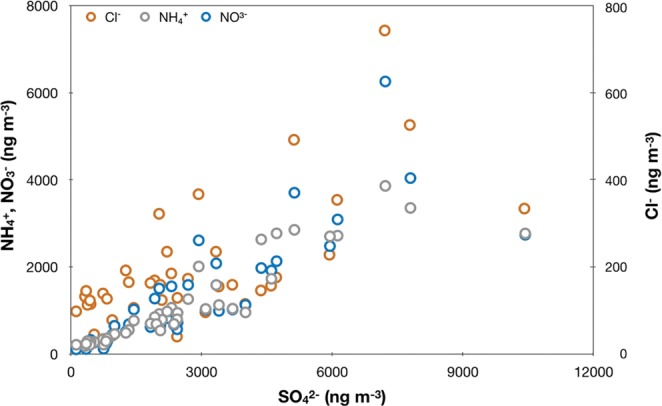
Scatter plots of Cl^−^, NO_3_^−^, NH_4_^+^ versus SO_4_^2−^. The respective R^2^ values are 0.47, 0.70, and 0.83, respectively. The *p*-values are all less than 0.001.

To assess sulfate originated from anthropogenic emission only we first remove the sea salt component following Hsu *et al*.^36^ Sea salt sulfate contributes little to the aerosol sulfate collected at Mt. Lulin. The contribution ranges from 0.2% to 2.7% maximum by mass, with an average of 1.2 ± 0.7%, further verifying the proposition that oceanic dimethyl sulfide is not a major source of sulfate at LLN. Correlation analysis shown in Fig. 2 demonstrates that the collected non-sea salt sulfates (nss-SO_4_^2−^) are largely affected anthropogenically. Tight correlation of [nss-SO_4_^2−^] with [NO_3_^−^] (R^2^ = 0.70) or [NH_4_^+^] (R^2^ = 0.83) suggests human activities play a major role in the production of sulfate aerosols in the atmosphere; the correlation with nitrate is expected as a result of high temperature combustion and the correlation with ammonium is via NH_3_ slipped from power plants. Anthropogenic origin of sulfate aerosols is also supported by statistically good correlation with [CO], with R^2^ = 0.36 and *p*-value = 2 × 10^–5^. Complete regression analysis (not shown here but analysis results supporting the statement are available in Guha *et al*.^37^) shows that statistically significant correlation is found for the gaseous (CO and O_3_) and aerosol-phase (ions) species considered, demonstrating anthropogenic alteration is a major source in affecting their abundances. The collected sulfates covering all seasons with little sea salt contribution suggest one may take the values of sulfates collected at the site to represent a regional background anthropogenic level in east Asia.

**Figure 3:**
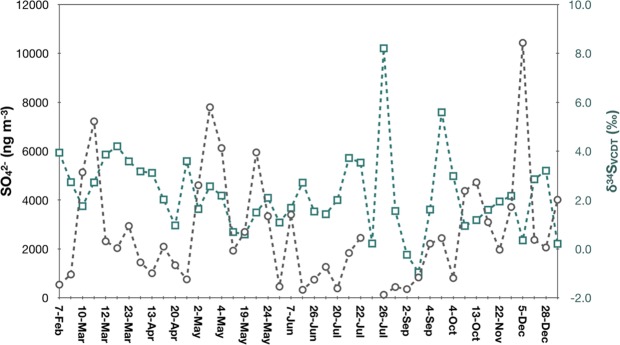
Time series of δ^34^S and SO_4_^2−^.

The δ^34^S values vary between −1.0 and 8.2‰ and are averaged to 2.2 ± 1.6‰ (Table 2). Unlike sulfate concentration (see also Guha *et al*.^37^), there is no observable seasonality (Fig. 3). The values in summer and the rest of the seasons are 2.1 ± 2.3‰ and 2.3 ± 1.3‰, respectively. We then analyze the data with aid from their air mass 5-day back trajectories obtained using NOAA ARL HYSPLIT4 model^41^ with the GDAS (Global Data Assimilation System) meteorological data provided by NCEP (National Center for Environmental Prediction) at a resolution of 6 hours in time and 190.5 km in horizontal spread (see Guha *et al*.^37^ for details) and divide the data into two categories (noted in Table 2): one tracks back to a lower region (lower than the sampling site altitude) of the atmosphere and near the surface (ocean surface exclusively) and the other one in regions higher than the sampling location; see Guha *et al*.^37^ for a thorough discussion and presentation on the origins of air masses. Similar to seasonal variations, no statistically significant difference is noted: the former is 2.5 ± 2.3‰ and the latter is 2.1 ± 1.0‰. Moreover, no statistically significant correlation is found for δ^34^S and other variables examined in this work, suggesting sulfur-bearing species have been processed physically and chemically many times attaining certain level of homogenization in space and time before turning into sulfate phase arriving at the sampling location. That is, the source characteristics have been lost, and the sulfur isotopes represent a regional average. The conclusion is supported by triple-oxygen isotope analysis made for sulfate at a background site in east Asia^42^.

We then compare with major regional sources of sulfur from China. The values of δ^34^S in sulfate aerosols in PM_2.5_ reported for Beijing, China during 2015 China Victory Day (with strict pollution control) and non-control periods are 4.7 ± 0.8‰ and 5.0 ± 2.0‰, respectively^43^. The corresponding concentrations are 3560 ± 2050 ng m^−3^ and 9590 ± 10910 ng m^−3^. For comparison, the level of sulfate at another strict control period, the 2008 Olympic, is even higher than the non-control period in 2015 mentioned above^7,44^. Indeed, strategic regulation help reduce pollution level but the outcome heavily depends on local/regional meteorology^7^. Overall, the 2015 control period gives sulfate ~50% greater than the value of LLN. The seasonal variations are apparent in the concentrations of the species reported in this work, but δ^34^S is not. From their two-year (2004–2005) of study in Japan, Sakata *et al*.^33^ showed that both the δ^34^S and concentration of sulfate varied seasonally. Heavier sulfate (that is, higher δ^34^S value) reported in winter time tends to be less abundant in the concentration, and they suggested the aerosols were originated in northern China^33,45^. From their analysis, elevated abundance in sulfate in summer time is related to petroleum combustion and has little to do with coal burning. The argument is supported by the δ^34^S values and vanadium concentrations in the collected aerosols and air mass back trajectory for the samples.

**Figure 4:**
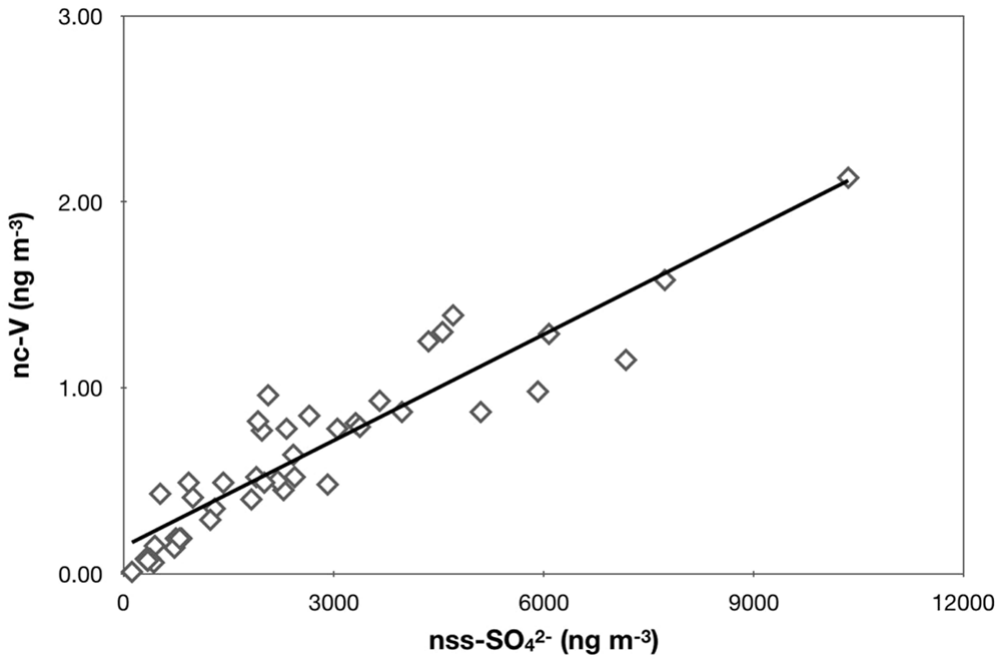
Scatter plot of nss-SO_4_^2−^ versus nc-V (noncrustal vanadium). See text for the calculation of noncrustal fraction. The R^2^ and *p*-values are 0.86 and <0.001, respectively.

The δ^34^S values measured in the aerosols collected at LLN are significantly lower than those reported in China^7,45–49^. Our values are in general close to the values obtained by Sakata *et al*. (2013) in summer time and to some degree, our results are in agreement with the values from a high mountain in southeast China, Mt. Wuyi in summer time^48,49^ when there is less influence from coal burning. Following the same analysis as Sakata *et al*.^33^, strong correlation between non-crustal vanadium (nc-V) and nss-SO_4_^2−^ concentrations is found (R^2^ = 0.86, *p*-value < 0.001; Fig. 4); crustal contribution is estimated using the V/Al ratios reported in Japan arc upper crust^50^. The overall crustal contribution is 12 ± 6%, with the summer time value (8 ± 6%) slightly less than the rest of the seasons (13 ± 6%). Both the current study and that of Sakata *et al*.^33^ suggest that a major source of sulfate in the east Asia is likely from oil industry, rather than coal burning. Evidence is also seen from the poorer correlation (R^2^ = 0.45, *p*-value < 0.001) between sulfate and lead. The core reason behind for the correlations is that emission from oil industry is enhanced in vanadium concentration and that from coal burning is lead-enriched (see Sakata *et al*.^33^ and references contained therein). Finally, we note that the non-seasonally varying sulfate δ^34^S values measured at LLN strongly suggests LLN can be a representative site for regional background sulfate. The regional contribution from coal industry, however, is yet to be determined and that is critically dependent on the source characteristics of sulfur-bearing compounds from oil industry which has not been quantified in east Asia.

## Concluding Remarks

We reported one-year sulfur isotope analysis for suspended sulfate aerosols collected at the high mountain station Lulin in the Western Pacific. Regardless of the origins of air masses, the δ^34^S values in the sulfates are averaged to 2.2 ± 1.6‰. No clear seasonality is seen, and the marine contribution for the sulfate loading is determined to be less than 3%. Time series analysis for the concentrations of lead and vanadium, however, does show significant enhancement in spring (March-June) and winter (September-December) time. The former is due clearly to biomass burning is southeast countries (e.g., see^37,51,52^). The latter is affected by winter monsoons that carry pollutants from China. Correlation analysis for sulfate with lead and vanadium shows that [SO_4_^2−^] correlates with vanadium (R^2^ = 0.85, *p*-value < 0.001) better than lead (R^2^ = 0.45, *p*-value < 0.001), suggesting oil industry plays a critical role in affecting sulfate level at Mt. Lulin. The results indicate that coal burning is less significant than oil industry but its contribution is yet to be determined. Despites the correlations observed and noted above, no statistically significant correlation is observed for δ^34^S with any of the physical quantities measured. The results imply that the sulfur-bearing species might have been processed many times before converting into sulfate aerosols and reaching the sampling location, with their source isotopic information greatly diminished. As a result, we suggest the δ^34^S values of Lulin sulfates can represent the level of the background in the Western Pacific. The average is 2.2 ± 0.2‰ (1 standard error, n = 44). This regional value is essential to quantitatively estimate the budget of sulfur in a local and even to a country-sized scale in Asian countries where fossil fuel burning affected air quality has been an issue of public concerns in the past decade and will likely remain in the coming decade.
